# Beyond Boundaries: Metastasis of Cervical Squamous Cell Carcinoma to the Kidney

**DOI:** 10.7759/cureus.87492

**Published:** 2025-07-07

**Authors:** Fadhel Yusuf, Fatema Yusuf, Amal Hayat, M. Zaid Jarai, Amr Elmekresh, Elham Mahjoor Azad, Fariborz Bagheri

**Affiliations:** 1 Urology, Dubai Hospital, Dubai, ARE; 2 Internal Medicine, Ibn Al-Nafees Hospital, Manama, BHR; 3 Urology, Dubai Health, Dubai, ARE; 4 Family Medicine, Dubai Health, Dubai, ARE

**Keywords:** cancer, cervical squamous cell carcinoma, kidney, metastasis, tumor recurrence

## Abstract

Cervical squamous cell carcinoma (SCC) is an aggressive gynecological malignancy that commonly metastasizes to the lungs, liver, and bones; renal involvement is extremely rare. We report the case of a 68-year-old woman with a history of high-grade cervical SCC, treated a decade earlier with hysterectomy and bilateral salpingo-oophorectomy, who recently presented with right flank pain and hematuria. Imaging revealed a mass involving the bladder and right distal ureter causing hydronephrosis. She underwent a cystoscopy with right ureteral stenting and transurethral resection of the bladder tumor (TURBT). Intraoperative examination revealed a mass in the right lateral vaginal wall.

Histopathological examination of the TURBT specimen revealed invasive high-grade non-papillary SCC infiltrating the bladder wall, with sparing of the urothelial mucosa. Immunohistochemistry confirmed human papillomavirus (HPV)-associated, Grade 3 (poorly differentiated) recurrent cervical SCC.

A dimercaptosuccinic acid (DMSA) scan demonstrated a non-functioning right kidney, and due to recurrent infections, she underwent a robotic-assisted right nephrectomy. Pathological examination of the renal specimen confirmed metastatic cervical SCC. This case illustrates a rare metastatic pathway of cervical cancer and underscores the importance of multidisciplinary evaluation in complex clinical presentations.

## Introduction

Cervical squamous cell carcinoma (SCC) is a significant gynecological malignancy with well-known aggressive behavior and potential for recurrence [1]. The most common metastatic sites for cervical SCC are the lungs, liver, and bones [2]. However, metastasis to the kidney is exceedingly rare, with very few cases reported in the literature [3-5].

Cervical cancer represents the most prevalent malignancy linked to human papillomavirus (HPV), with nearly all cases, around 99.7%, resulting from ongoing infection with high-risk genital HPV strains [5]. Management of advanced cases typically involves a multimodal approach including chemotherapy and radiotherapy, yet treatment outcomes remain unfavorable, particularly in cases of recurrence or metastasis [6,7].

## Case presentation

**Figure 1:**
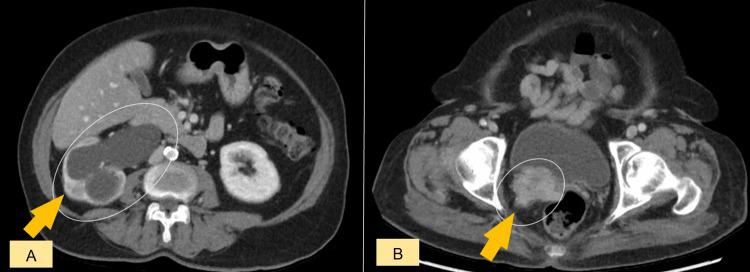
Contrast-enhanced CT scan of the chest, abdomen, and pelvis (axial views). (A) Right-sided hydronephrosis (arrow) caused by distal ureteric obstruction from the underlying mass. (B) Irregular mass (arrow) in the rectovesical pouch infiltrating the right posterior bladder wall and distal ureter, resulting in right hydroureteronephrosis.

A 68-year-old female patient with a surgical history of hysterectomy and bilateral salpingo-oophorectomy for cervical cancer performed 10 years ago presented to the emergency room with right flank pain and hematuria. A contrast-enhanced CT scan of the chest, abdomen, and pelvis revealed an irregular mass in the rectovesical pouch, infiltrating the right posterior bladder wall and distal ureter, causing hydronephrosis (Figure 1). Pulmonary nodules were also detected, raising suspicion of metastatic disease.

The patient was admitted as an inpatient and underwent cystoscopy with the placement of a 7 French, 26 cm right ureteral tumor stent, followed by transurethral resection of the bladder tumor (TURBT). Intraoperative pelvic examination revealed an irregular, hard mass in the right lateral vaginal wall. Histopathological analysis of the bladder tumor specimen demonstrated invasive high-grade non-papillary SCC infiltrating the bladder wall and muscle while sparing the urothelial mucosa, suggesting extension from adjacent tissues. Immunohistochemistry (IHC) of the TURBT specimen showed tumor cells positive for P63, CK5/6, CK19, CK7, and p16, and negative for GATA-3, CDX2, CK20, synaptophysin, chromogranin, CD56, and uroplakin. These findings confirmed the diagnosis of HPV-associated, Grade 3 (poorly differentiated) recurrent cervical SCC.

**Figure 2:**
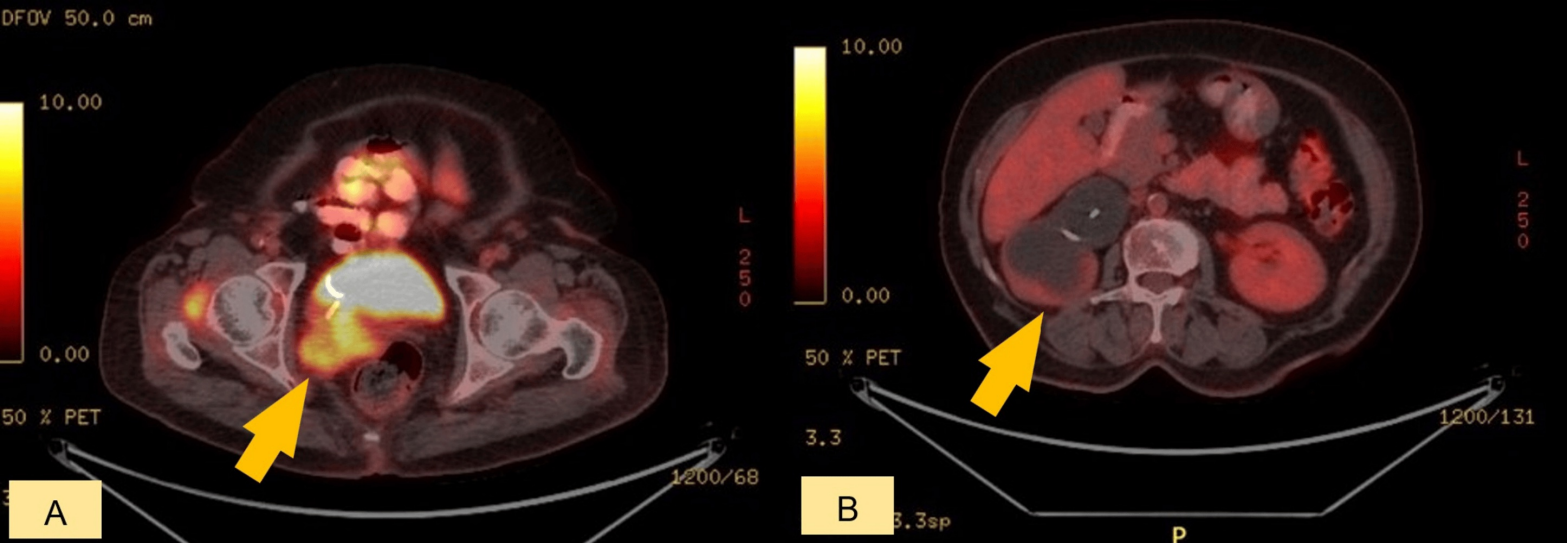
Fluorine-18 fluorodeoxyglucose positron emission tomography-computed tomography (F-18 FDG PET-CT). (A) Hypermetabolic mass (arrow) in the pelvic floor, extending from the vagina to the base of the urinary bladder and right ischiorectal region, correlating with a poorly differentiated squamous cell carcinoma. (B) PET-CT image showing right-sided hydronephrosis (arrow) due to distal ureteric obstruction.

Over the following months, multiple investigations, including a fluorine-18 fluorodeoxyglucose positron emission tomography-computed tomography (F-18 FDG PET-CT) from the skull base to mid-thigh, indicated hypermetabolic activity in the pelvic floor, extending from the vagina to the base of the urinary bladder and right ischiorectal region, correlating with poorly differentiated SCC (Figure 2).

Metastatic involvement of the right internal iliac lymph nodes, right ischium, and right lung was suspected, along with significant right-sided hydronephrosis with an in situ right ureter stent. No surgical intervention was deemed curative, and the patient was started on chemotherapy.

**Figure 3:**
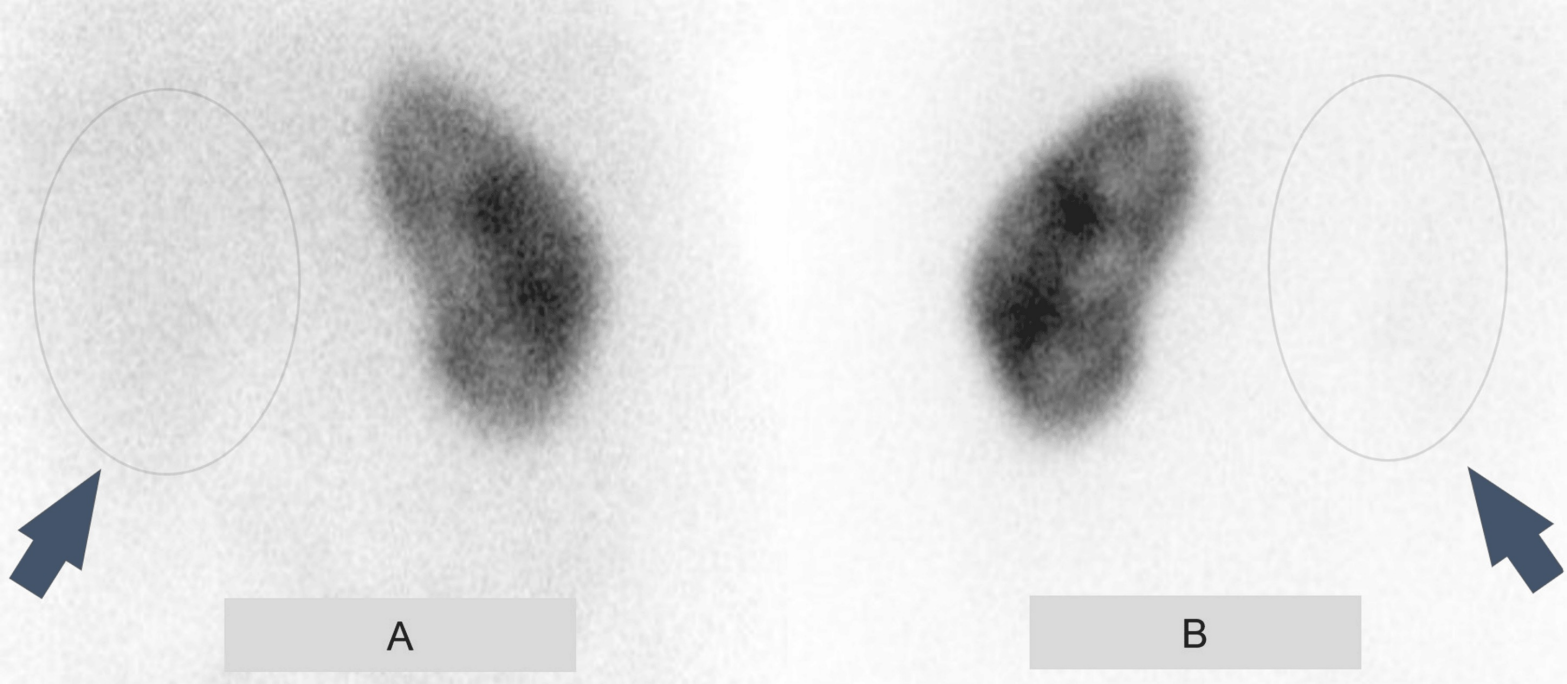
Dimercaptosuccinic acid (DMSA) renal scan demonstrating a non-functioning right kidney. (A) Anterior view showing the absence of function in the right kidney (arrow). (B) Posterior view showing non-function of the right kidney (arrow), with preserved cortical function of the left kidney.

Subsequent imaging showed a decrease in the size of the pelvic mass, though it continued to infiltrate the bladder, ureter, cervix, and upper vagina, resulting in the development of a vesicovaginal fistula (VVF) causing persistent urine leakage. The patient underwent multiple ureteral stent replacements due to persistent obstruction and the development of an atrophic right kidney. A dimercaptosuccinic acid (DMSA) renal scan indicated a non-functioning right kidney with good cortical function of the left kidney (Figure 3).

The patient then underwent a robotic-assisted right simple nephrectomy. Histopathology of the renal specimen revealed deposits of poorly differentiated SCC measuring approximately 1.3 cm, which had infiltrated the surrounding adipose tissue. The pelvic muscle wall and urothelium appeared lesion-free. Additionally, the kidney tissue exhibited signs of chronic pyelonephritis and interstitial nephritis.

Following nephrectomy, the patient developed a VVF, resulting in continuous urine leakage. Her general condition deteriorated, prompting a multidisciplinary discussion, which concluded that immediate surgical repair of the VVF was inadvisable. A PET scan was recommended to evaluate for further metastatic disease.

Despite the risks, an attempt to repair the VVF was made, but her condition remained complicated by persistent leakage and progressive malignancy. Subsequent MRI imaging revealed a stationary mass in the vaginal wall and bladder wall thickening, with no significant interval changes.

The patient was later admitted with signs of small bowel obstruction. Exploratory laparotomy confirmed loco-regional recurrence of malignancy in the pelvis, complicated by a frozen pelvis. The surgery revealed infiltration of the terminal ileum with metastatic disease, causing stricture, as well as the involvement of the distal jejunum and proximal ileum by metastatic nodules, leading to bowel narrowing and dense adhesions. She was managed surgically and discharged with a functioning ileostomy. During a follow-up oncology visit, the patient reported fatigue and being bedridden, and was advised to focus on supportive care and maintaining a healthy lifestyle.

## Discussion

Cervical SCC remains one of the most common gynecological malignancies worldwide [1], predominantly caused by persistent infection with high-risk HPV, particularly types 16 and 18 [6]. The most common sites of distant metastasis of cervical cancer are the lungs, bone, and liver [8], while the less frequent locations of spreading are the bowel, adrenal gland, spleen, and brain [9].

However, metastasis to the kidney is exceedingly rare and not well characterized in the literature. Few case reports and small series have described renal involvement secondary to cervical SCC, emphasizing the atypical nature of this metastatic pattern and the diagnostic challenges it presents [3,4].

The pathophysiology underlying renal metastasis from cervical SCC is not fully understood but is hypothesized to involve hematogenous dissemination or retrograde spread through the lymphatic system and urinary tract [5]. When renal metastasis does occur, it is important to distinguish it from primary renal malignancies, particularly urothelial carcinoma, through careful histopathological evaluation.

Histopathological examination supplemented by IHC is essential to differentiate metastatic cervical SCC from primary renal tumors. Histopathological confirmation of metastatic involvement may be established through biopsy or examination of nephrectomy specimens [4]. In the present case, immunohistochemical analysis demonstrated strong overexpression of the p16 protein, supporting a cervical origin of the metastatic lesion. p16 is commonly upregulated in cervical squamous epithelium due to the oncogenic activity of the E7 protein expressed by high-risk HPV strains. This overexpression is observed in approximately 95% of invasive cervical carcinomas, rendering p16 a reliable surrogate marker for HPV-associated cervical malignancies [10].

Due to the rarity of renal metastasis from cervical SCC, there is limited consensus on optimal diagnostic and therapeutic strategies. However, documenting and studying such cases contributes valuable insight into the metastatic behavior of cervical cancer, guiding clinicians in recognizing and managing these atypical presentations.

## Conclusions

This case report highlights the exceptionally rare occurrence of cervical SCC metastasizing to the right kidney, an uncommon and often underrecognized metastatic route. It contributes valuable insight into the spectrum of atypical disease progression and emphasizes the critical role of a multidisciplinary approach in managing complex oncologic cases.

## References

[1] Jain MA, Limaiem F (2023). Cervical squamous cell carcinoma. StatPearls [Internet].

[2] Zhou S, Peng F (2020). Patterns of metastases in cervical cancer: a population-based study. Int J Clin Exp Pathol.

[3] Rodriguez J, Castro JC, Beltran M, Forero O, Pareja R (2019). Simultaneous metastasis from cervical cancer to the kidney and paraspinal muscle: a case report. Cureus.

[4] Bazine A, Zniber HO, Ghaouti M, Bazine A, Baydada A, Sifat H (2017). An uncommon case of renal metastasis from cervical cancer. Cureus.

[5] Kulkarni MM, Khandeparkar SG, Joshi AR, Kothikar V (2016). A rare case of renal metastasis from squamous cell carcinoma of the cervix. J Midlife Health.

[6] Okunade KS (2020). Human papillomavirus and cervical cancer. J Obstet Gynaecol.

[7] Shen Y, Meng X, Wang L, Wang X, Chang H (2022). Advanced primary vaginal squamous cell carcinoma: a case report and literature review. Front Immunol.

[8] Dai Y, Zhang Y, Ke X, Liu Y, Zang C (2023). Cutaneous metastasis from cervical cancer to the scalp and trunk: a case report and review of the literature. J Med Case Rep.

[9] Cohen PA, Jhingran A, Oaknin A, Denny L (2019). Cervical cancer. Lancet.

[10] Klaes R, Friedrich T, Spitkovsky D (2001). Overexpression of p16(INK4A) as a specific marker for dysplastic and neoplastic epithelial cells of the cervix uteri. Int J Cancer.

